# Supplementary material for: Diversity and dynamics of the CRISPR-Cas systems associated with Bacteroides fragilis in human population
Source: BMC Genomics. 2022 Aug 11;23:573. doi: 10.1186/s12864-022-08770-8 (PMC9367070; doi:10.1186/s12864-022-08770-8)
Supplement: Supplementary file 1 — Additional file 1 Additional file 1: Supplementary information. Supplementary Figure 1 shows the heatmap visualization of the spacer-MGE network and Supplementary Figure 2 shows the heatmap visualization of the host-MGE network. [file 12864_2022_8770_MOESM1_ESM.pdf]

Supporting Information

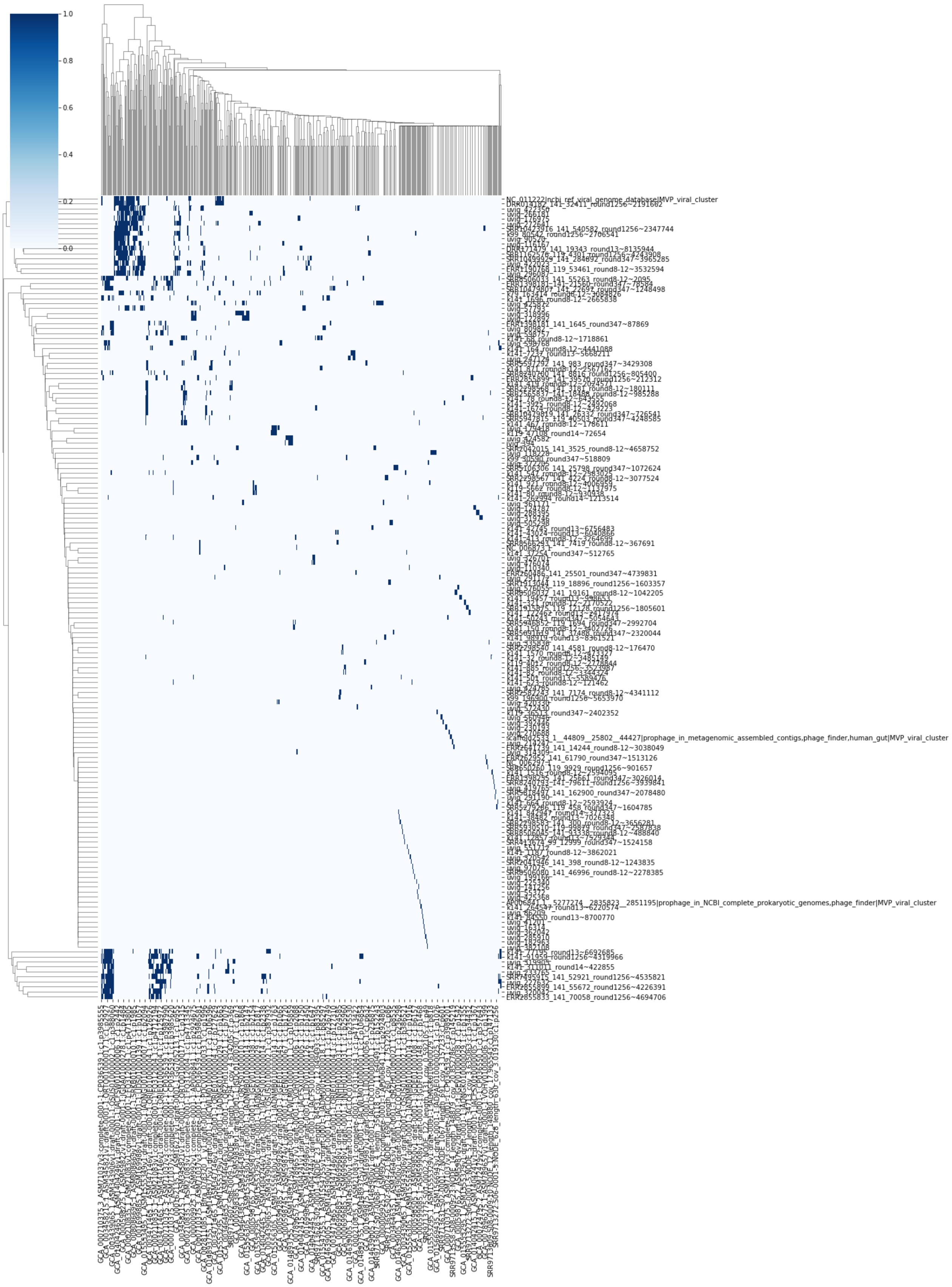

Figure S1: The heatmap visualization of the spacer-MGE network. The MGEs are shown along y-axis and the spacers are shown along x-axis. A cell is shown in blue if the corresponding MGE contains a protospacer matching the corresponding spacer.

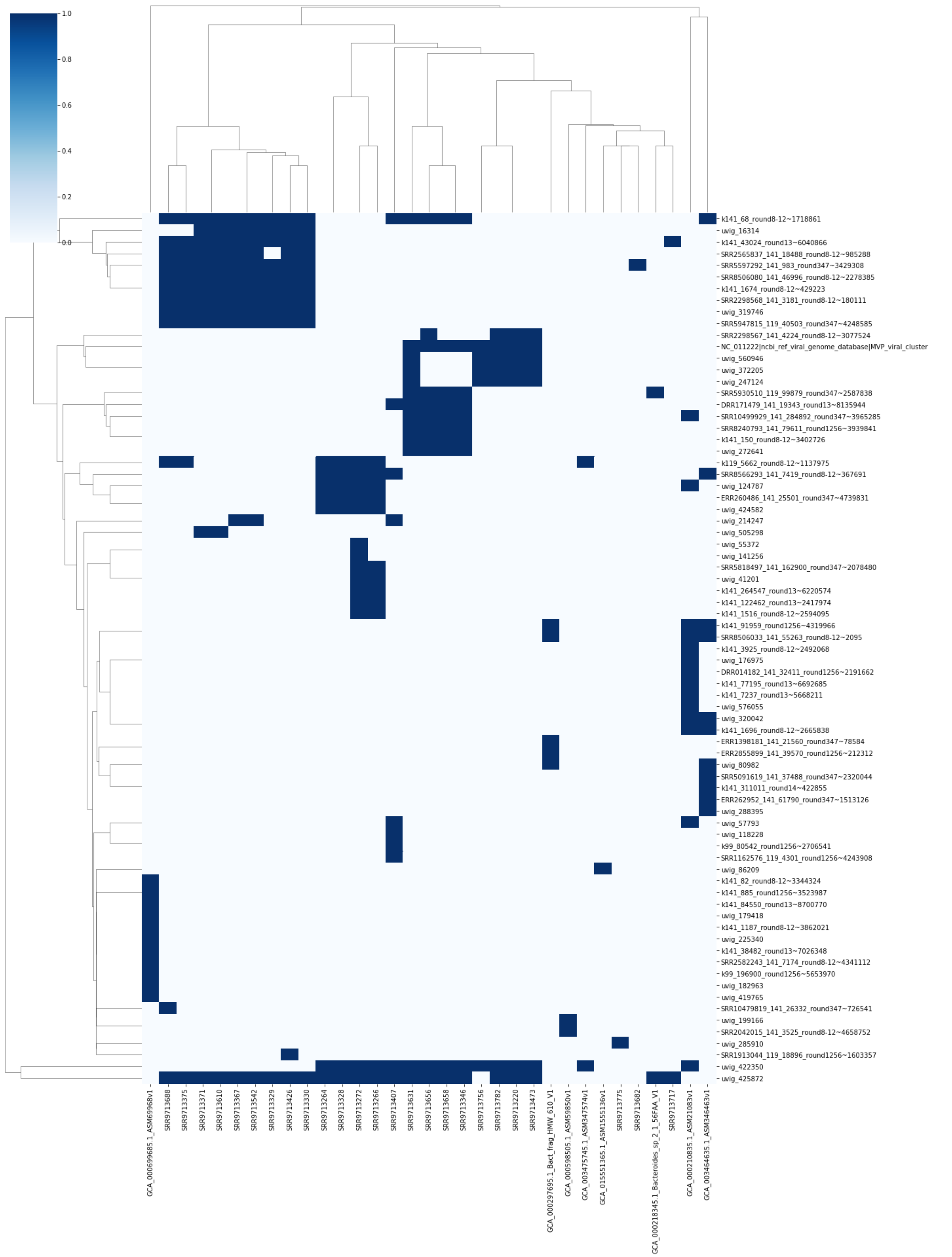

Figure S2: The heatmap visualization of the host-MGE network. The MGEs are shown along y-axis and the hosts are shown along x-axis. A cell is shown in blue if the corresponding MGE contains a protospacer matching a spacer found in the corresponding host's CRISPR array.
